# Fellow-Workers and the Roll of Honour

**Published:** 1915-01-16

**Authors:** 


### ROUND THE WORLD.

[The Editor Invites Early Information and Contributions Marked "Fellow-Workers."]

### Fellow-Workers and the Roll of Honour.

Dr. Samuel West, who has been appointed consulting physician to the new King George Hospital at Waterloo, occupies a similar position at St. Bartholomew's and the Royal Free Hospitals, as well as at the New Hospital for Women. Educated at Christ Church, Oxford, and subsequently at "Bart.'s," Dr. Samuel West displayed brilliant qualities at the very outset of his career, and becoming attached to the staff of his hospital after much hard work, built up for himself a very solid reputation as a consultant. Apart from his many writings on general medicine, his works on diseases of the chest are, perhaps, best known to practitioners in this country. It may be recalled to mind that their author was in earlier years physician to a well-known chest hospital—namely, the City of London Hospital at Victoria Park. A censor and member of the Council of the Royal College of Physicians, as well as examiner in medicine for various universities and president of several learned societies, this distinguished clinician has occupied many of the foremost positions open to our leading physicians.

Of the two members of the staff of St. Bartholomew's Hospital who have lately received military promotion, Lieutenant-Colonel Archibald E. Garrod is physician and lecturer on chemical pathology at that institution. A brilliant investigator of physiological and pathological problems, he was made an F.R.S. for his work, whilst some of his more important conclusions have been published in the Bradshaw and Croonian lectures of the Royal College of Physicians. An M.D. Oxon., F.R.C.P. Lond., and honorary LL.D. Aberd., Colonel Garrod married the eldest daughter of the late Sir Thomas Smith, the famous surgeon. He worked for many years at the Great Ormond Street Hospital for Sick Children, to which he is now consulting physician.

Lieutenant-Colonel H. J. Waring, the colleague in promotion of the last-named, is surgeon and joint lecturer in surgery at St. Bartholomew's Hospital. An M.S., B.Sc. Lond., F.R.C.S. Eng., Colonel Waring, who originally came to "Bart.'s" from Owens College, Manchester, is the author of a work on "Surgical Diseases of the Liver, Gall-Bladder, and Biliary System," as well as of a popular "Manual of Operative Surgery." Formerly an examiner in surgery for London University, he now examines for the Royal College of Surgeons, and is a consulting surgeon to the Metropolitan Hospital. Colonel Waring is also to be congratulated on his appointment as one of the surgeons to the new King George Hospital at Waterloo.

Major George Newton Pitt, one of the eight physicians appointed to the King George Hospital, for many years accomplished brilliant work at Guy's Hospital, to which institution he is now consulting physician. An M.D., B.C. Cantab., he became an F.R.C.P. Lond. in 1889, and delivered the Gulstonian lecture for the Royal College of Physicians in the following year. As a young man he did well at Cambridge, his name appearing tenth on the list of Wranglers, whilst he was a Fellow of Clare College from 1880 to 1886. Subsequently becoming elected to the staff at "Guy's," he closely associated himself with the advancement of scientific medicine there, and has published many important papers.

Mr. F. Newland-Pedley, who has been appealing from Rouen for an organised staff of dentists, is a well-known West-End dental surgeon, and one of the consultants attached to the dental department at Guy's Hospital, which he actively served for many years. At the outbreak of war Mr. Newland-Pedley went to the Continent and volunteered his services to the military authorities. Apparently he was largely in requisition from the outset, and quickly formed the opinion that many men might be kept in the Army if adequate arrangements were made for the care of their teeth. It should be noted that at the time of the South African war Mr. Newland-Pedley went to the Front as a member of the staff of the Imperial Yeomanry Hospital, and so has had ample experience of military requirements in regard to his specialty. A student of "Guy's," Mr. Newland-Pedley became an L.D.S. in 1880, and qualified as a surgeon in the following year, subsequently becoming an F.R.C.S. Eng. At one time he was dental surgeon to the Evelina Hospital for Children and to the old North-West London Hospital.

Sir Warren Roland Crooke-Lawless, M.D., M.Ch. R.U.I., who took over the command of the British Red Cross Hospital at Netley, has had a distinguished career in the Army Medical Service, which he joined soon after qualifying in the 'eighties. Subsequently he was appointed to the Coldstream Guards, in which regiment he was given a commission as surgeon-major a few years later. He served with distinction through the South African war, being then promoted to his present rank of surgeon-lieutenant-colonel. Sir Warren Crooke-Lawless's services brought him many honours, including a knighthood and the C.I.E., before he retired three years ago. He has for many years made his home in Ireland, where he was for some time surgeon to his Excellency the Viceroy, and is now J.P. for the County of Cork.

Major Henry M. W. Gray, R.A.M.C.(T.), who has been doing excellent work as chief surgeon to the British Hospital at Wimereux, is a prominent Aberdeen surgeon, and holds the posts of surgeon to the Royal Infirmary and to the Royal Hospital for Sick Children in the famous old Scottish University city. Qualifying M.B., C.M. Aberd., in 1895, Major Gray also studied extensively on the Continent, and attended the London Hospital for some time. Eventually taking the F.R.C.S. Edin., he settled down to surgical practice, and, in spite of much active work, has found time to write largely on operative and general surgical technique.

Mr. A. H. Rabagliati, who is one of the assistant surgeons working under Major Gray at Wimereux, is an M.D., Ch.B. Edin. and M.R.C.S. Edin. A Fellow of the Obstetrical Society of Edinburgh, he has had much clinical experience, having held the appointments of surgeon to the Ingham Hospital, Queensland; house surgeon to the Women and Children's Hospital at Leeds; and house surgeon to the Royal Maternity and Simpson Memorial Hospital at Edinburgh.

